# Supplementary material for: The interactome of intact mitochondria by cross-linking mass spectrometry provides evidence for coexisting respiratory supercomplexes
Source: Mol Cell Proteomics. 2017 Dec 8;17(2):216–32. doi: 10.1074/mcp.RA117.000470 (PMC5795388; doi:10.1074/mcp.RA117.000470)
Supplement: Supplemental Data [file supp_RA117.000470_133922_0_supp_23978_fzffwf.zip › spectra_annotation/mito_spectra_annotation/29-1-22-1-10-1.pdf]

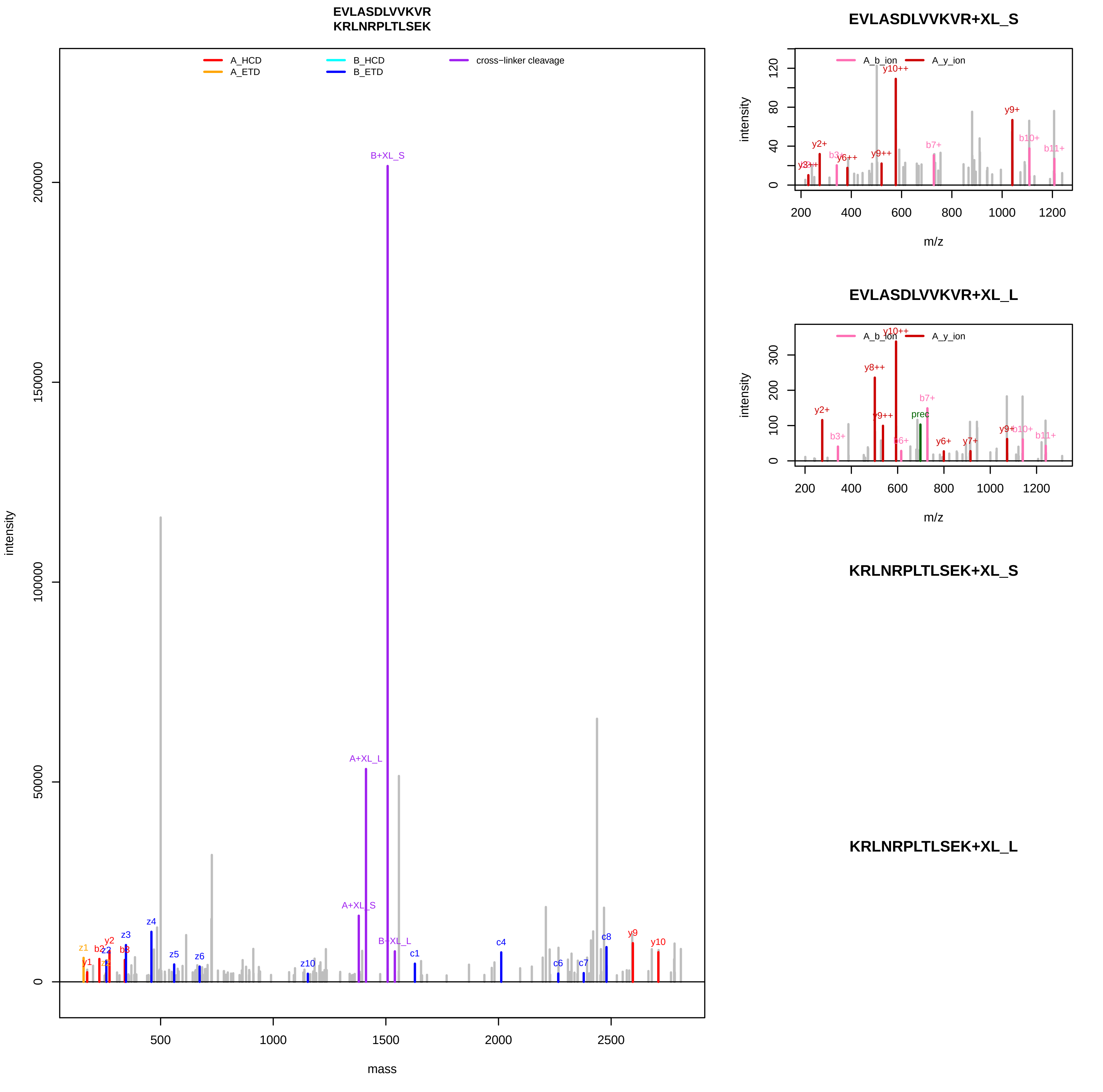
EVLASDLVVKVR
KRLNRPLTLSEK
A_HCD
A_ETD
B_HCD
B_ETD
cross−linker cleavage
B+XL_S
A+XL_L
A+XL_S
B+XL_L
z1
y1
b2
z2
y2
z3
b3
z4
z5
z6
z10
c1
c4
c6
c7
c8
y9
y10
intensity
mass
0
50000
100000
150000
200000
500
1000
1500
2000
2500
EVLASDLVVKVR+XL_S
A_b_ion
A_y_ion
y10++
y9+
y2+
b3+
y6++
y9++
b7+
b10+
b11+
intensity
m/z
0
40
80
120
200
400
600
800
1000
1200
EVLASDLVVKVR+XL_L
A_b_ion
A_y_ion
y10++
y8++
b7+
y2+
y9++
prec
b3+
b6+
y6+
y7+
y9+
b10+
b11+
intensity
m/z
0
100
200
300
200
400
600
800
1000
1200
KRLNRPLTLSEK+XL_S
KRLNRPLTLSEK+XL_L
